# Pausing behavior of late bilingual and monolingual English speakers

**DOI:** 10.1016/j.heliyon.2023.e21322

**Published:** 2023-10-27

**Authors:** Abdullah A. Alfaifi, Hussain Almalki, Yuting Guo, Zhiyan Gao

**Affiliations:** a King Abdulaziz University, College of Science and Arts, Department of English, POB 344, Rabigh, 21911, Saudi Arabia; b George Mason University; c Jiangsu Shipping College, English Department, Nantong, Jiangsu Province, 226009, China

**Keywords:** Pausing, Lexical complexity, Syntactic complexity, Prosody, Speech rate, Second language acquisition

## Abstract

This study examined the differences in the pausing behavior between native and non-native English speakers. Specifically, it examined the location and duration of pauses in relation to the syntactic and lexical complexity of the clauses in which these pauses occur and the nature of the prosodic phrasing of the utterances containing pauses. Speech samples from 10 native (L1) English and 10 Mandarin non-native English speakers from the Archive of L1 and L2 Scripted and Spontaneous Transcripts and Recordings (ALLSSTAR) were included in the analysis. The results showed that lower-level prosodic boundaries and syntactically complex phrases were associated with significantly longer pause duration in the L2 speech. Additionally, phrases with less frequent words tended to induce longer pauses. These findings suggest that insufficient knowledge of the L2 syntax, lexicon, and prosody might determine the location and duration of pauses and ultimately affect the speech fluency of L2 speakers.

## 1 Introduction

One of the primary differences between native and non-native speakers is fluency, which is the ability to produce meaningful strings of words uninterruptedly [1]. Fluency is generally viewed as the level of proficiency one has with a language. It has been shown that this ability relies heavily on underlying cognitive processing [[2], [3]]. and requires a high degree of automaticity, a skill that native speakers have [4]. This automaticity is understood in terms of speech rate and duration of pauses, among other competencies [5]. Fluent speech, hence, requires substantial linguistic knowledge and native-like speech planning routines. The lack of such linguistic knowledge in the speech of L2 speakers could be due to insufficient linguistic knowledge of the L2 [6]. Insufficient linguistic knowledge is often referred to as incomplete acquisition of the L2, resulting in decreased fluency in L2 speech.

The differences between native and non-native speech fluency are reflected in many linguistic domains. Specifically, non-native speakers generally differ from native speakers in their speech rate, prosodic planning, lexical access, and syntactic processing [[7], [8], [9], [10], [11]]. Previous research has shown that the L2 lexicon is generally smaller and less complex than the L1 lexicon, which implies difficulty in both lexical access and processing [10]. Additionally, L2 speakers are generally slower in tasks like naming pictures [12], and syntactic and lexical complexity have also been found to influence speech production in L2 speakers’ speech production [11,13]. L2 speakers generally use simple structures and vocabulary and have difficulty producing more complex structures. Significant differences in speech rate and prosodic planning have been found between native and non-native speakers in spontaneous and read utterances [8,9,14].

Thus, incomplete acquisition is expected to affect speech production and perception, which will be more pronounced among L2 speakers with weaker linguistic knowledge when compared to native speakers. This study focused on pausing as one area that has been found to distinguish L1 and L2 speakers. Previous research has shown that non-native speakers have longer and more frequent pausing behaviors than native speakers [8]. Therefore, it is predicted that the pausing behavior will interact with speech rate, prosodic phrasing, and lexical and syntactic complexity. To this end, we hypothesized that incomplete acquisition would be reflected as a difference in pausing between native and non-native speakers. Since L2 speakers have insufficient knowledge of the L2, we expected their pauses to be longer and occur more frequently in the L2 speech. We also expected a relationship between the pauses and one or more of the linguistic domains we examined.

We also expected that L2 speakers would have a slower speech rate than native speakers, which could also increase the duration of their pauses. Furthermore, due to the insufficient knowledge of prosodic phrasing in L2, we expected the L2 speakers to require more planning time, which often results in long pauses. Additionally, the degree of lexical and syntactic complexity in the L2 requires longer planning and processing time and, thus, longer pause duration.

This study investigated the relationship between pausing behaviors in L2 (English) reading and contextual complexity (i.e., lexical and syntactic complexity) in controlled prosodic environments. The results provide evidence for the effect of complexity on information processing and speech fluency. The following section describes the relevant research that motivated the present study.

## 2 Background

### 2.1 Syntactic complexity

Pauses are associated with the syntactic constituent's hierarchy. For example [15], reported that in reading tasks performed by native speakers, pauses generally occurred at syntactic boundaries and were longer than other types of pauses. Additionally, the more complex the syntactic structure, the longer the pause [15]. These findings imply that non-native speakers will exhibit even longer pauses than native speakers, given their limited level of proficiency in the L2.

Measures of syntactic complexity have ranged from simple to complex. [16]; for instance, identified the mean length of utterances as a reliable measure of syntactic complexity. Although the mean length of utterance seems insensitive to structural differences, the assumption is that longer utterances are inherently more complex since they require more processing time and are likely to include more complex structures.

Hierarchal relations between syntactic constituents in utterances have also been proposed to be a reliable predictor of syntactic complexity. Utterances with embedded clauses are syntactically more complex than simple utterances [17,18]. Utterances with embedded clauses are then more difficult to process and subsequently exhibit more and longer pauses [[19], [20], [21], [22]]. [[23], [24]] reported that pauses are likely to precede subordinate and embedded clauses in French and English, with the latter being less frequent. Pauses also occur at sentence boundaries and before content words [25].

Different types of utterances and clauses with varying complexity require different linguistic planning and processing [26,27]. Accordingly, a relationship between the relative complexity of the phrase and the occurrence and duration of pauses is expected [28,29]. Furthermore, the location of the pauses could either be before the complex structure, which is a result of planning, or after the complex structure, which is a matter of processing [30].

### 2.2 Lexical complexity

Lexical complexity is another factor that affects the pause duration in L2 speakers. Lexical complexity is defined as lexical richness, sophistication, variation, or density [31]. However, in this study, lexical complexity was determined by examining how frequently a word occurs in L2 (English). Since more complex tasks in L2 require more language processing [32], the hypothesis was that lexically more complex phrases would require more language processing and, thus, may influence the speaker's fluency and pause duration in the L2. Therefore, we asked whether pauses by L2 speakers while reading aloud would be influenced by lexical complexity (i.e., word frequency). Previous research on high- and low-frequency words in the L2 and the related eye movements (tracking and fixation) while reading has shown that high-frequency words are accessed and recognized faster in the L2 and have fixations with a shorter duration than low-frequency words.

For instance, in a reading task [33], compared L1 and L2 frequency effects in word recognition by Dutch/English bilinguals and English monolinguals. They found that the bilinguals' responses were faster for L1 (Dutch) words than for L2 words (English). They also found that responses were faster for high-frequency words than for low-frequency words for both monolinguals and bilinguals. Eye movement and fixation studies have also shown that high-frequency words receive shorter fixation time than low-frequency words [34].

Based on this robust finding, it was expected that low-frequency words would correspond with longer fixations—and possibly longer pause durations—which is argued to be caused by slower lexical access for low-frequency words [35]. Therefore, phrases with less frequent words were expected to result in durational differences in pauses between non-native and native speakers. Depending on the location of the pause, this could be explained in terms of planning (if pauses occur before phrases with low-frequency words) or processing (if pauses occur after phrases with low-frequency words). The durational differences, if found, may be attributed to the L2 speakers' incomplete lexical acquisition in their L2. Thus, the hypothesis for lexical complexity is that pauses associated with low frequency words will be longer since the reader will have to assign a prioritization to produce that lexical item accurately and access it in planning and processing. As a result, it was predicted that lexical complexity can be an important factor affecting pause durations and speech rate when the speaker is reading text aloud.

### 2.3 Prosodic phrasing

With pre-boundary lengthening and pitch change, pausing is an acoustic correlate of prosodic boundaries [36,37]. These acoustic cues at prosodic boundaries are fundamental to successful language comprehension because they often coincide with syntactic constituents and are instrumental in influencing syntactic parsing [38]. Although both adults and children are sensitive to boundary cues, research on L1 speech shows that not all cues carry the same weight in language comprehension. German speaking adults, for example, are more sensitive to pitch change and pre-boundary lengthening than to pausing [39]. On the other hand, younger children do not show sensitivity to pausing at all, while older children with fully developed syntactic abilities are sensitive to pausing as a boundary cue [40].

These findings show that the implementation of pausing as a boundary cue relies heavily on one's syntactic abilities. Thus, the pausing behavior of second language learners with less-developed syntactic abilities is likely to diverge from that of native speakers. For instance, L2 speakers of English pause more frequently and have more extended periods of silence in their L2 speech than native speakers [41]. Although pausing seems less critical in L1 speech perception, it is vital to foreign accent detection, even more important than stress timing and peak alignment [41,42]. [43] further showed that pausing is more reliable in predicting perceived foreign accents than speech rate. However, previous research often overlooks the differences between pausing as a boundary cue and pausing as a signal for disfluency. While longer pausing often arises from the syntactic and lexical complexity of the context, the question remains as to whether the incomplete acquisition of L2 prosody can be reflected in pause duration in the L2 speech.

In addition to boundary-induced pauses, speech rate is another factor that affects pausing patterns [44]. argue that non-native speech is usually slower than native. Therefore, non-native pausing is expected to be longer than native pausing if the speech rate and pausing duration are proportional. Furthermore, besides slower speech rate, read speech by non-native speakers exhibits more significant speech rate variability than native read speech [7]. Based on this observation, the local speech rate is more likely to be associated with the preceding and following utterances than the entire speech rate.

Although contextual complexity (syntactic and lexical) has been studied in different areas, there is a gap in the literature concerning the relationship between pausing and incomplete acquisition represented in the syntactic and lexical domains. This study contributes to the literature by examining the precise relationship between pausing and incomplete acquisition in read speech.

## 3 Methodology

### 3.1 Materials

The materials of this study were collected from the Archive of L1 and L2 Scripted and Spontaneous Transcripts and recordings (ALLSSTAR; [45]. In this archive, native and non-native English speakers read "The North and the Sun" passage in English. Accordingly, we extracted speech samples from ten Mandarin/English bilinguals and ten native English speakers to analyze for this study. The read speech was chosen because it provided an environment where contextual complexity and prosodic phrasing could be relatively controlled.

### 3.2 Analysis

Each read speech sample was divided into nine phases, according to the punctuations in the script, and eight pausing locations were identified. This strategy was adopted because punctuations indicate the levels of prosodic boundaries, especially in read speech [46]. In this study, commas were considered signs of lower-level boundaries, while periods were considered signs of higher-level boundaries. The reading material was as follows where "||" marks higher level boundaries, and "|" marks lower-level boundaries, yielding four higher-level and four lower-level prosodic boundaries.

"The north wind and the sun were disputing which was the stronger, | when a traveler came along wrapped in a warm cloak. || They agreed that the one who first succeeded in making the traveler take his cloak off should be considered stronger than the other. || Then the north wind blew as hard as he could, | but the more he blew the more closely did the traveler fold his cloak around him; | and at last the North Wind gave up the attempt. || And then the sun shined out warmly, | and immediately the traveler took off his cloak. || And so the North Wind was obliged to confess that the sun was the stronger of the two."

For the acoustic analysis, we used Praat [47] to manually mark boundaries between the end of a phrase and the beginning of a pause and boundaries between the end of the pause and the beginning of the following phrase. Then, we used a Praat script to measure the duration of all the marked pauses automatically and produced a spreadsheet containing the durational information of pauses for further analysis.

This study used "the number of embedded clauses" as the measurement for syntactic complexity and the mean logarithmic (log) word frequency for lexical complexity. Syntactic complexity was measured using the L2 syntactic complexity analyzer web application [48]. Lexical complexity was measured by calculating the logarithmic mean of log word frequencies for all content words within each phrase. The log word frequency data was taken from the Lexile framework for reading [49].

During the statistical analysis of processing complexity, processing complexity was defined as the syntactic and lexical complexity before the pauses. To investigate the effect of syntactic complexity on pause duration, we constructed linear mixed effects models in R [50] with the lme4 package [51]. Syntactic complexity (i.e., the number of embedded clauses per phrase) preceding the pauses, the level of prosodic boundaries (low vs. high), the participant's native language (L1 background), were used as independent variables, along with the interactions between these variables. Pause duration was the dependent variable. Subjects were entered as a random effect with syntactic complexity, the level of prosodic boundary, and their interaction term as the random slope. Since all participants paused at the eight boundaries mentioned above, and the pauses at these locations might be associated with adjacent words, the eight locations of the pauses were treated as items and were subsequently a random effect.

Finally, in order to investigate whether the contextual complexity of the phrase that followed a pause had an effect on pause duration, we constructed linear mixed effects models with pause duration as the dependent variable. The fixed effects were syntactic complexity following pauses (i.e., the number of clauses within the phrase immediately following a pause), L1 background, level of prosodic boundaries, and the interactions between the three. Subjects were entered as a random effect with syntactic complexity, boundary level, and their interactions as the random slope. Pause location was also entered as a random effect.

## 4 Results

### 4.1 Complexity processing

**Fig. 1:**
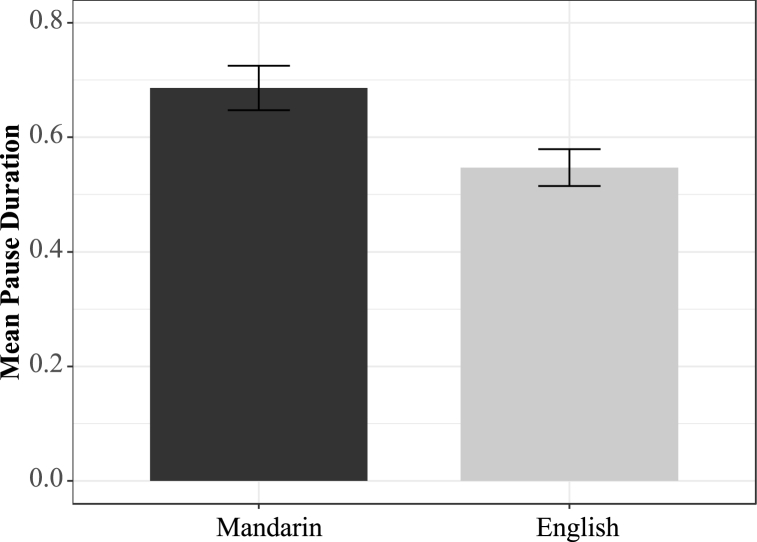
Mean Pause Duration (s) by L1 background.

On average, Mandarin/English late bilinguals paused for 686 ms (SD = 346 ms), which was longer than native English speakers' average pause duration (M = 546 ms, SD = 286 ms). Fig. 1 demonstrates the pausing duration for Mandarin speakers *(black bar)* and English speakers *(grey bar)*, where the error bars represent a 95 % confidence interval.

**Fig. 2:**
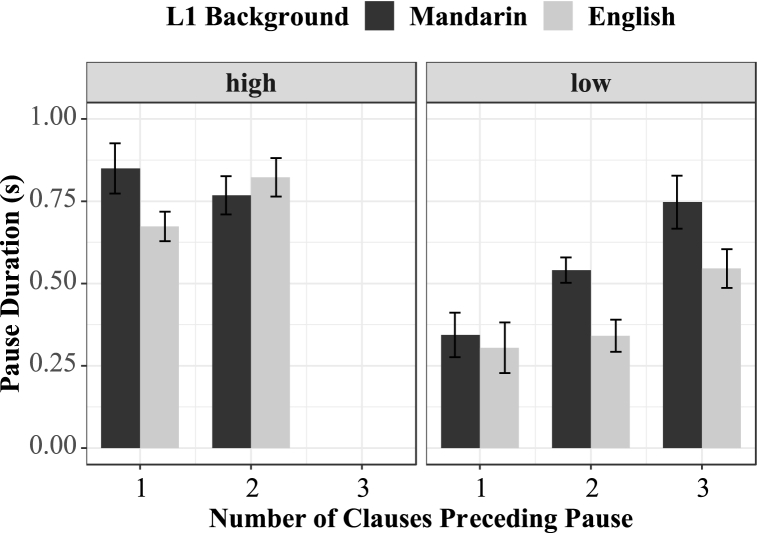
Mean pause duration at different levels of prosodic boundaries.

In order to investigate the effect of syntactic complexity on pause duration, we constructed linear mixed effects models (see 3.2 for more details). Model comparisons using the Likelihood Ratio Test showed that L1 background did not significantly contribute to model fit (χ^2^ = 0.88, p = .93), suggesting that Mandarin/English bilinguals do not necessarily differ from native English speakers in terms of pausing duration. The level of prosodic boundary (low vs. high) significantly contributed to model fit (χ^2^ = 19.578, p < .001), with lower-level boundaries inducing shorter pauses. The interaction between prosodic boundary and L1 background did not significantly contribute to model fit (χ^2^ = 3.46, p = .06), suggesting that both Mandarin speakers and English speakers paused longer at higher-level boundaries and shorter at lower-level boundaries. The results revealed a significant interaction between L1 background, the level of prosodic boundary, and syntactic complexity (χ^2^ = 4.97, p < .05), showing that syntactic complexity only affected pausing duration on lower-level prosodic boundaries, and the effect was more prominent to the bilinguals than to native English speakers. Fig. 2 demonstrates the mean pause duration at different levels of prosodic boundaries, where the black bar represents the Mandarin speakers and the grey bar represents the English speakers.

Mean log frequency was later added to the models as a fixed effect to investigate whether lexical complexity preceding the pause would affect its duration. However, mean log frequency did not significantly contribute to the model fit, nor were there any interactions between mean log frequency and other fixed effects. Interestingly, as measured by mean log frequency, lexical complexity positively correlated with pause duration (r = 0.34), demonstrating a possible effect of lexical complexity on pause duration. However, no definitive conclusions can be drawn at this point.

Speech rate—the number of syllables per second—was also added to the models. In this analysis, the speech rate was separately measured for each phrase by dividing the duration of a phrase by the number of syllables it contained. The number of syllables and the duration of each phrase were first identified with a Praat script [52]. Two trained linguists later manually corrected them.

To investigate whether the variability of speech rate affected pause duration, we added the preceding speech rates (i.e., speech rates of phrases that precede pauses) to the models. Model comparisons showed no significant effect of speech rate on pause duration. Pause duration might also be proportional to the length of its preceding phrases. To investigate the effect of phrase duration on pause duration, we entered the length of the phrase (i.e., phrase length that precedes a pause) into the model as a fixed effect. However, no significant effect of phrase duration was found.

### 4.2 Complexity planning

**Fig. 3:**
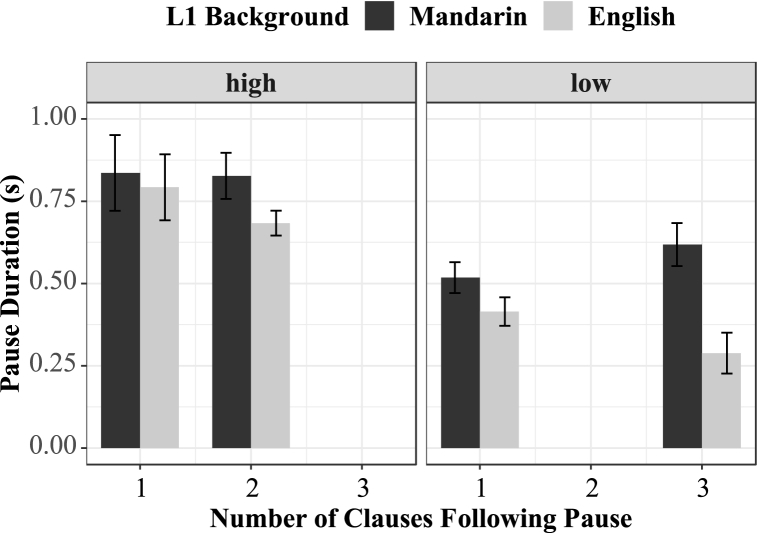
Mean pause duration and Syntactic complexity following pauses.

Pause duration could be associated with planning time. Readers might quickly scan through the following phrase before they start reading it. Therefore, the contextual complexity of the phrase that follows a pause could also affect pause duration. To investigate whether this hypothesis was correct, we constructed linear mixed effects (see 3.2 for more details). Model comparisons using the Likelihood Ratio Test revealed no significant effects. The level of prosodic boundaries achieved marginal significance (χ^2^ = 9.91, p = .07). Higher prosodic boundaries induced longer pauses. Mean log frequency and the interactions between mean log frequency, L1 background, syntactic complexity, and boundary levels were added to the model fixed effects later. Again, no significant effects were observed. Fig. 3 demonstrates the interaction between prosodic boundaries and syntactic complexity following a pause.

## 5 Discussion

Late bilinguals (Mandarin/English) were less fluent in the reading task than monolingual English speakers, as demonstrated by the overall pause duration difference between the two groups. Additionally, the interaction between pause duration, lower-level prosodic boundary, and complex syntactic structure was significant, indicating a lack of fluency resulting from the incomplete syntactic acquisition of the L2.

The difference in pause duration at syntactic boundaries was assessed by comparing late bilinguals and monolinguals in a reading task. It was revealed that late bilinguals paused longer after syntactically more complex phrases associated with lower-level prosodic boundaries. Syntactic complexity was defined in terms of how many embedded clauses were in the phrase. Processing needs could drive this durational effect on the pauses. That is, due to the lack of fluency in the L2, late bilinguals presumably require a longer time to process the previous complex phrase and a shorter time to process less-complex phrases. Such an interaction was not observed for higher-level prosodic boundaries, perhaps because pauses at higher-level prosodic boundaries are generally longer, providing enough time for the processing to be completed. Under this interpretation, the syntactic complexity, lower-level prosodic phrases, and information processing may be the determinant factors for the pause duration.

No significant differences in pause duration before syntactically complex phrases were found between groups. It is possible that since this was a reading task, the reader did not require more time for planning before complex phrases because the phrases were available to them.

The results from pause duration after the complex syntactic structure are consistent with previous studies that have assessed the correlation between pause duration and syntactic complexity [30,53]. The significant findings from this study show that the more complex the preceding and following syntactic constituents, the longer the pause duration. These findings are relevant to Ref. [54] findings. They reported that the complexity of the previous phrases determines the boundary strength and subsequently results in a longer duration more often than the complexity of the upcoming phrase.

As for lexical complexity, measured by log word frequency, the results were not significant. However, in the interaction between pause duration and lexical complexity, the higher the number of complex words in a phrase, the longer the pause. Further analysis and studies should examine other criteria besides frequency when measuring lexical complexity. Word length, for instance, can be a criterion since other studies have shown that longer words have longer fixations in reading [55]. Analyses on the relationship between speech rate and pause duration were not significant, suggesting that pausing behaviors might be independent of speech rate in reading tasks.

This study did not consider L1 pausing patterns and participants' L2 proficiency. It is likely that Mandarin speakers’ pausing behavior observed in this study is simply a result of the negative transfer from L1 prosody. On the other hand, as discussed in previous research, pausing patterns in the L2 might be influenced by pausing patterns in the L1 and the speakers' overall L2 proficiency [56]; Levelt, Roelofs & Meyer, 1999). A speech production model proposed by Ref. [57] postulates that L1 speech production is correlated with L2 speech production. This hypothesis was further confirmed by studies investigating the relationship between L1 and L2 fluency in spontaneous speech [58,59]. [60] examined the relationship between L2 proficiency and pausing in both L1 (Russian) and L2 (English) in spontaneous speech. Her results showed that highly proficient L2 speakers could produce native-like pause patterns, which suggests that the transfer effect of L1 pausing patterns can be overcome as L2 proficiency improves.

## 6 Conclusion

This paper used native and non-native English read speech to assess pause duration in the L2. Pause duration was explored in relation to syntactic and lexical complexity, prosodic phrasing, and speech rate. The results showed that lower-level prosodic boundaries and syntactic complexity were associated with longer pause duration in L2 speech. We interpreted this interaction as a processing reflex. In addition, phrases with less-frequent words tended to induce longer pauses. These findings suggest that insufficient knowledge of the L2 syntax, lexicon, and prosody might interact with pause location and duration. Given the findings from this study, it is important to consider pedagogical approaches that address the issue of pause duration and fluency in L2 learners. For instance, working on the recognition of syntactic boundaries can help late bilinguals better recognize complex phrases, which could then lead to fewer pauses at lower- and higher-level prosodic boundaries. Additionally, strategies such as self-monitoring and awareness of pauses during production can help learners become more fluent in their speech. It could be beneficial to provide (late) bilinguals with exercises that focus on recognizing syntactic boundaries as well as the length and timing of their pauses in order to improve their fluency. Furthermore, focusing on lexical complexity and word length should also be considered when designing language learning activities. These strategies could be used in the classroom to help students become more fluent and increase their L2 proficiency. Future research should also consider how prosody affects other language tasks such as speaking and writing, as well as the correlation between L2 proficiency and fluency. Examining these areas will provide insights into language learning techniques that can alleviate the issue of pause duration for L2 learners and enhance fluency. Finally, it is important to consider that late bilinguals may have different needs when compared to early or advanced bilinguals and, thus, may require a different approach in language instruction. Taking these factors into consideration can help create more effective learning methods for (late) bilinguals.

## 7 Limitations of the current study

The pausing patterns in L1 and the L2 proficiency of the participants were not fully explored in this study, and these are areas in which future work can explore. The observed pause behavior among Mandarin speakers in this study could primarily be due to the adverse transfer from L1 prosody. However, based on existing studies, we know that L2 pausing patterns could be affected by both the pausing tendencies in L1 and the overall L2 proficiency of the speakers as discussed above.

Another potential constraint of this study lies in its focus on read speech when examining the relationship between pausing and incomplete language acquisition. Exploring this relationship within the context of spontaneous speech might lead to more comprehensive and accurate insights, thereby enriching the validity of the results. This shift in focus would more closely emulate real-world language use, potentially yielding a more authentic understanding of language acquisition processes. This is another prominent area of research for future work to explore.

## Data availability statement

Data will be made available on request.

## CRediT authorship contribution statement

**Abdullah Ahmad Alfaifi:** Conceptualization, Formal analysis, Funding acquisition, Methodology, Project administration, Resources, Supervision, Validation, Writing – original draft, Writing – review & editing. **Hussain Almalki:** Conceptualization, Data curation, Formal analysis, Methodology, Software, Validation, Writing – original draft, Writing – review & editing. **Yuting Guo:** Conceptualization, Data curation, Software, Validation. **Zhiyan Gao:** Conceptualization, Formal analysis, Investigation, Methodology, Software, Supervision, Validation, Visualization, Writing – original draft.

## Declaration of competing interest

The authors declare that they have no known competing financial interests or personal relationships that could have appeared to influence the work reported in this paper.
